# Supplementary material for: TSPO PET Imaging as a Potent Non-Invasive Biomarker for Diffuse Intrinsic Pontine Glioma in a Patient-Derived Orthotopic Rat Model
Source: Int J Mol Sci. 2022 Oct 18;23(20):12476. doi: 10.3390/ijms232012476 (PMC9603997; doi:10.3390/ijms232012476)
Supplement: Supplementary file 1 [file ijms-23-12476-s001.zip › ijms-1961988-supplementary.pdf]

Supplementary materials

TSPO PET imaging as a potent non-invasive biomarker for Diffuse Intrinsic Pontine Glioma in a patient-derived orthotopic rat model

Céline Chevaleyre[1], Dimitri Kereselidze[1], Fabien Caillé[1], Nicolas Tournier[1], Nagore G. Olaciregui[2], Alexandra Winkeler,[1] Xavier Declèves[3], Benoit Jego,[1] Salvatore Cisternino[3,4], Sylvain Auvity[1,3,4,+], Charles Truillet[1,*,+]

[1]Université Paris-Saclay, CEA, CNRS, Inserm, BioMaps, Service Hospitalier Frédéric Joliot, 4 place du général Leclerc, 91401, Orsay France.
[2]Institut de Recerca, Sant Joan de Déu Hospital, Barcelone, Spain
[3]Université Paris Cité, Inserm UMR-S 1144, Paris, France.
[4]Hôpital Universitaire Necker-Enfants Malades, Assistance Publique des Hôpitaux de Paris, AP-HP, F-75015 Paris, France.
[*] Corresponding authors: Charles TRUILLET: +33 1 69 86 77 27, charles.truillet@universite-paris-saclay.fr, 91400
[+] both authors contributed equally to this work.

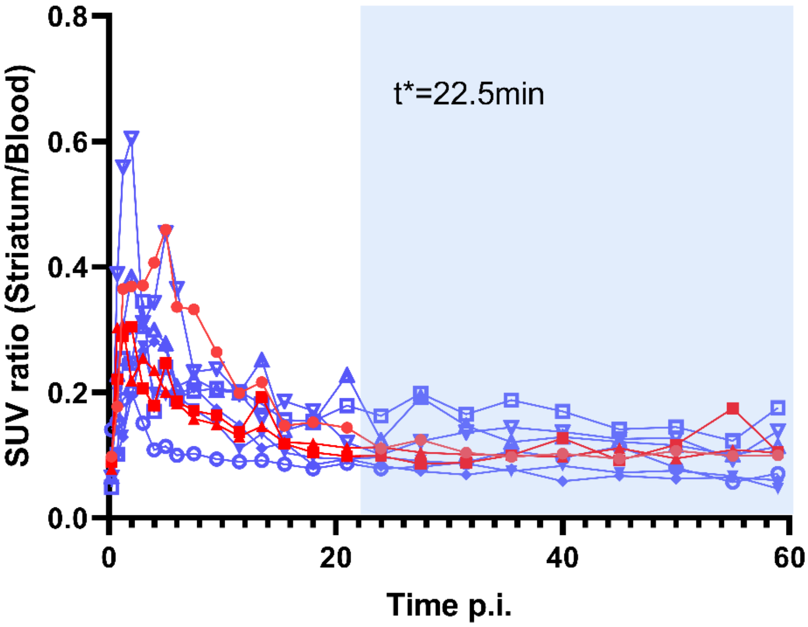

Figure S1. Striatum to blood ratio kinetic of ^{18}F-DPA-714. The steady state is reached at 22.5 min post injection. Individual data of the DIPG group are shown in blue, and data of the sham group are shown in red.

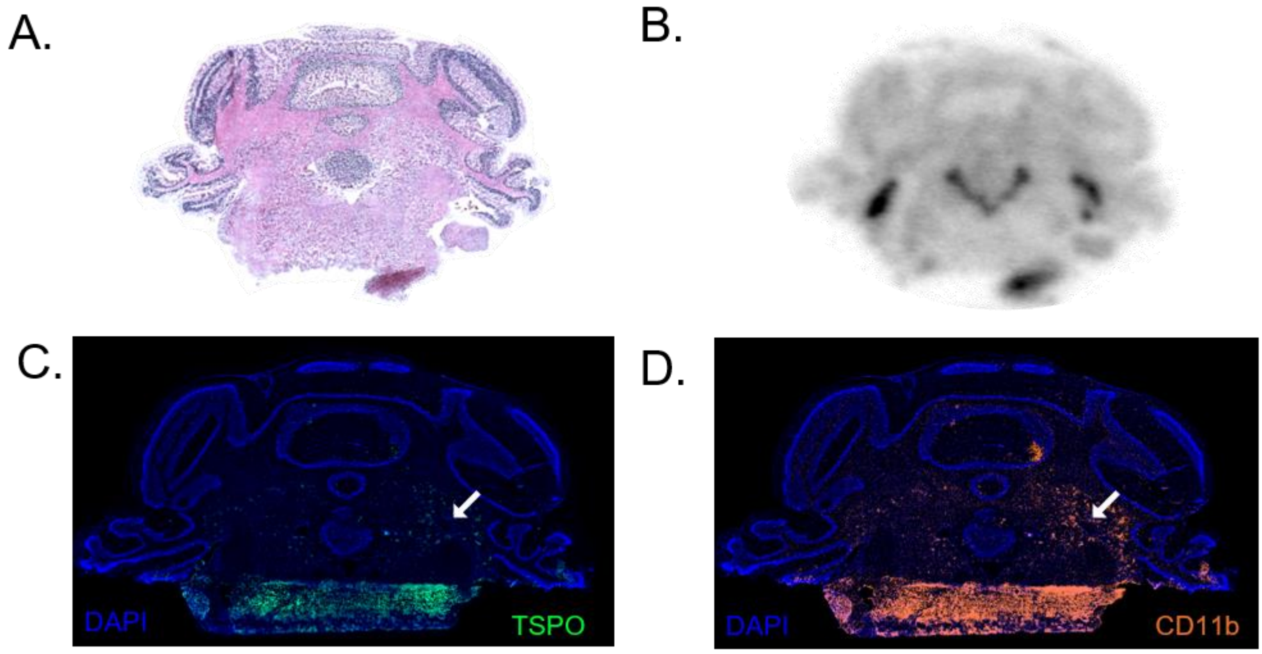

Figure S2. TSPO expression within the pons area in the sham group. (**A**) H&E staining, (**B**) autoradiography in vitro after [18F]-DPA-174 incubation on the same brain slice, (**C**) TSPO immunofluorescence, (**D**) CD11b immunofluorescence on adjacent brain slices. White arrows indicate the injection area. High intensity signals on the bottom of the slices (**C** and **D**) are unspecific signals due to the folding of the brain tissue.

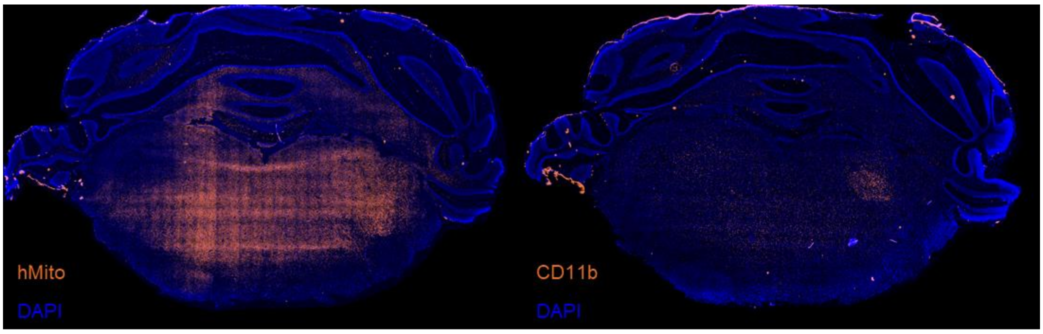

Figure S3. Correlation between DIPG007 cells and microglial cells localization. DIPG007 cells are stained with an antibody targeting the human mitochondria (hMito) and activated microglial cells are identified by the expression of CD11b.

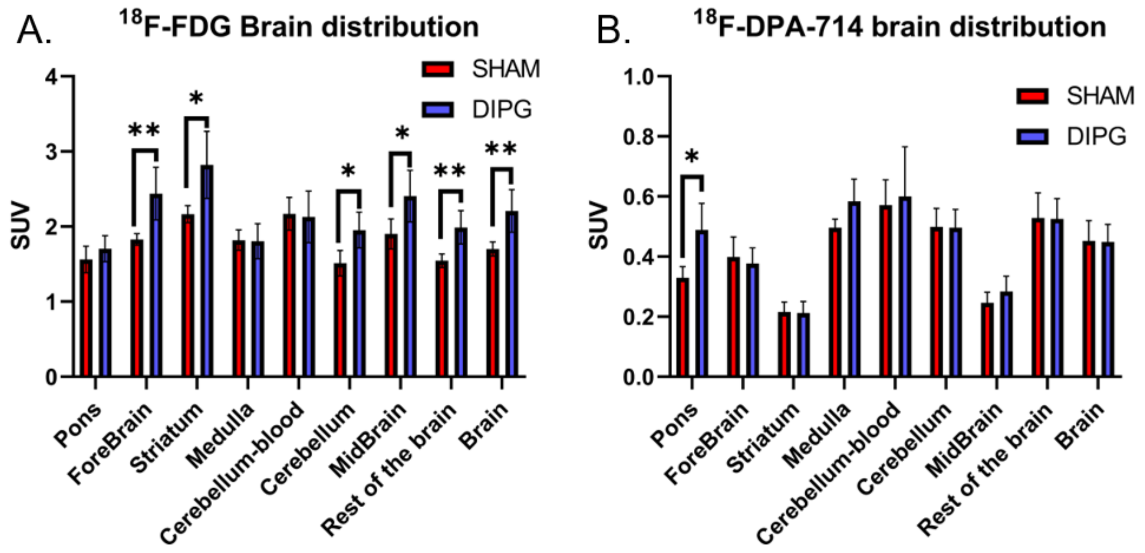

Figure S4. Brain distribution of ^{18}F-FDG (**A**) and ^{18}F-DPA-714 (**B**). Statistical significance was determined using *t*-test with * $p < 0.05$, ** $p < 0.01$. All data are represented as mean ± SD.

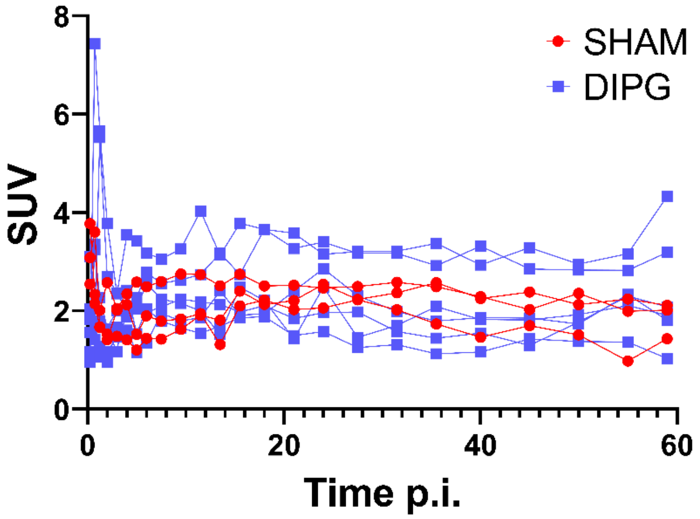

Figure S5. Individual blood time activity curves of ^{18}F-DPA-714 injected rats. Individual data of the DIPG group are shown in blue, and data of the sham group are shown in red.

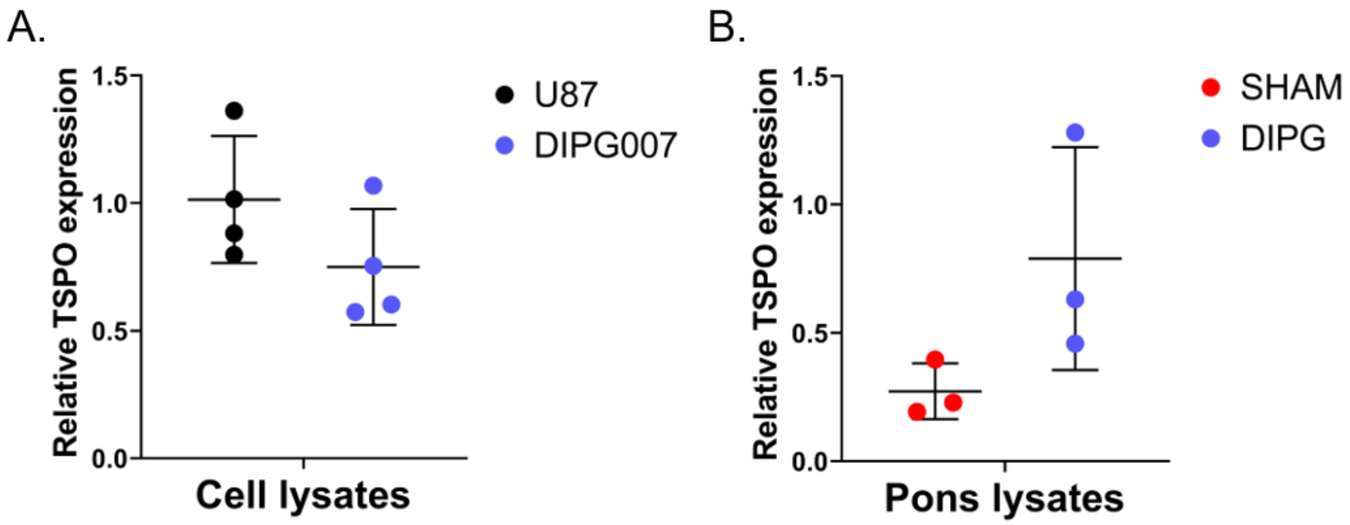

Figure S6. Relative expression of TSPO quantified by Western Blot. (**A**) *In vitro* DIPG007 and U87 TSPO expression (*n* = 4 replicates). (**C**) *Ex vivo* TSPO expression in the back brain of sham and DIPG007 cell-bearing rats (*n* = 3 replicates). TSPO expression was determined from the band intensity using ImageJ software, and normalized relative to the α-tubulin expression in the same sample. Mean ± SD is overlaid on individual measures.
